# Communication Impairment in Ultrasonic Vocal Repertoire during the Suckling Period of *Cd157* Knockout Mice: Transient Improvement by Oxytocin

**DOI:** 10.3389/fnins.2017.00266

**Published:** 2017-05-17

**Authors:** Olga L. Lopatina, Kazumi Furuhara, Katsuhiko Ishihara, Alla B. Salmina, Haruhiro Higashida

**Affiliations:** ^1^Department of Basic Research on Social Recognition and Memory, Research Center for Child Mental Development, Kanazawa UniversityKanazawa, Japan; ^2^Department of Biochemistry, Medical, Pharmaceutical, and Toxicological Chemistry, Krasnoyarsk State Medical University Named after Prof. V.F. Voino-YasenetskyKrasnoyarsk, Russia; ^3^Department of Immunology and Molecular Genetics, Kawasaki Medical SchoolKurashiki, Japan

**Keywords:** *CD157*, Bst-1, communication, development, ultrasonic vocalization, oxytocin, autism

## Abstract

Communication consists of social interaction, recognition, and information transmission. Communication ability is the most affected component in children with autism spectrum disorder (ASD). Recently, we reported that the *CD157/BST1* gene is associated with ASD, and that CD157 knockout (*Cd157*^−/−^) mice display severe impairments in social behavior that are improved by oxytocin (OXT) treatment. Here, we sought to determine whether *Cd157*^−/−^ mice can be used as a suitable model for communication deficits by measuring ultrasonic vocalizations (USVs), especially in the early developmental stage. Call number produced in pups due to isolation from dams was higher at postnatal day (PND) 3 in knockout pups than wild-type mice, but was lower at PNDs 7 and 10. Pups of both genotypes had similarly limited voice repertoires at PND 3. Later on, at PNDs 7 and 10, while wild-type pups emitted USVs consisting of six different syllable types, knockout pups vocalized with only two types. This developmental impairment in USV emission was rescued within 30 min by intraperitoneal OXT treatment, but quickly returned to control levels after 120 min, showing a transient effect of OXT. USV impairment was partially observed in *Cd157*^+/−^ heterozygous mice, but not in *Cd157*^−/−^ adult male mice examined while under courtship. These results demonstrate that *CD157* gene deletion results in social communication insufficiencies, and suggests that CD157 is likely involved in acoustic communication. This unique OXT-sensitive developmental delay in *Cd157*^−/−^ pups may be a useful model of communicative interaction impairment in ASD.

## Introduction

There is an increasing number of patients with syndromes of multiple etiologies that are covered by the umbrella of autism spectrum disorder (ASD) (Mychasiuk and Rho, 2016) Communication ability and emotional expression are major issues in children with ASD (Eigsti et al., 2011; DiStefano et al., 2016). Language communication from an early life stage is an essential tool for bidirectional information transmission (Fitch et al., 2010; Kuhl, 2011), and is clearly delayed in certain ASD subtypes.

Interdisciplinary research platforms (including non-human models) are interested in finding relevant pharmacological treatments (Shen et al., 2016; Wei et al., 2016; Zheng et al., 2016). In this regards, oxytocin (OXT) is a promising therapy. OXT is a neuropeptide with potent and profound effects on many physiological processes in the brain, cardiovascular system, and reproductive system (Gimpl and Fahrenholz, 2001). Specific pattern of OXT function (Zik and Roberts, 2015) directly relates to characteristics of behavior (Veenema and Neumann, 2008). Lower endogenous OXT levels are associated with impaired social cognition in various mental diseases including ASD, schizophrenia, and anxiety (Hoge et al., 2008; Zhang et al., 2012; Eapen et al., 2014; Strauss et al., 2015; Husarova et al., 2016; Massey et al., 2016). Further, OXT treatment rescues social behavioral deficits in animals (Jin et al., 2007; Freeman et al., 2016; Lawson et al., 2016; Teng et al., 2016), and patients with ASD during clinical trials to test its beneficial effects (Munesue et al., 2010; Althaus et al., 2016; Kosaka et al., 2016; Yatawara et al., 2016). We recently reported that under a social context, mutual interaction is significantly increased during the OXT arm of nasal administration, compared with the placebo arm, for ASD patients with intellectual disabilities (Munesue et al., 2016).

Behavioral changes are likely to be associated with brain OXT levels. OXT release from the soma, dendrites, and axon terminals of hypothalamic neurons occurs in response to intracellular Ca^2+^ mobilization (Amina et al., 2010; Lopatina et al., 2010; Leng et al., 2015; Higashida, 2016). This mechanism is regulated by activity of ADP-ribosyl cyclase CD38, a multifunctional molecule that combines enzymatic and receptor properties, and plays a key role in OXT secretion, critically regulating maternal and social behavior in mice (Jin et al., 2007; Salmina et al., 2010; Lee, 2011; Mushtaq et al., 2011; Schmid et al., 2011). Moreover, this mechanism is suggested to affect human behavior during development and in adulthood (Munesue et al., 2010).

In a context of CD38 as a social behavior regulator, the same gene family, bone marrow stromal cell antigen-1 (BST-1), also attracts attention as a similar social behavior regulator. BST-1 was first isolated from bone marrow stromal cell lines (Kaisho et al., 1994), and *BST1* was identified as *CD157* by gene cloning (Itoh et al., 1994). Interestingly, despite the important role of CD157 in the immune system (Shimaoka et al., 1998; Lo Buono, 2014), the *CD157/BST1* gene was identified as a risk-factor for neurodegeneration, particularly for Parkinson's disease (PD), or at least one of a variety of PD symptoms (Satake et al., 2009; Simón-Sánchez et al., 2011; Sharma et al., 2012; Zhu et al., 2012; Liu et al., 2013b). In addition, a new role for CD157 has been reported in stem cells, which is that CD157 induces the catalysis of cyclic ADP-ribose in paneth cells, which promotes intestinal stem cell self-renewal in mice that are on a calorie-restricted diet (Yilmaz et al., 2012), and cyclic ADP-ribose/CD157 promotes the proliferation of lung stem/progenitor cells (Wu et al., 2013). However, though very recently CD157 immunoreactivity has been shown to colocalize with nestin-positive cells in the ventricular zone in the brain (Higashida et al., 2017), little is known about the role of CD157 in brain function or in the brain degeneration deficits of PD.

Recently, there have been reports that the *CD157/BST1* gene is associated with other diseases, including ASD (Ceroni et al., 2014; Yokoyama et al., 2015). In addition, we have reported that CD157 knockout (*Cd157*^−/−^) mice display severe anxiety-related and depression-like behaviors or social avoidance that were reversed upon treatment with anti-depression drugs and OXT (Lopatina et al., 2014; Mizuno et al., 2015; Higashida et al., 2017). These findings prompted us to examine if the *Cd157*^−/−^ mice could be a suitable model of ASD or autistic-like behavior with impaired social behavior in the absence of motor dysfunction, especially in an early stage of development that has not been previously studied in these knockout mice.

Thus, the aim of our present study was to investigate the effect of *Cd157*/*Bst1* gene deletion on developmental aspects of vocal communication ability to determine if ASD-related communication deficits are due to a general impairment or developmental delay. Additionally, *Cd157* knockout mice are a useful ASD mouse model from the viewpoint of ASD-related communication impairment or delay. Accordingly, we show that *Cd157* knockout pups display a poor vocal repertoire (less variety in vocal pattern) during neonatal stages (postnatal day (PND) 3–10). Vocal syllable repertoire was reversely increased after 30–60 min of intraperitoneal injection of OXT in knockout pups at PND 7 and 10, although OXT did not advance the variety of vocal pattern. Finally, we show that the number of ultrasonic vocalization (USV) emissions is positively correlated with ADP-ribosyl cyclase activity and plasma OXT concentrations in wild-type but not knockout mice.

## Materials and methods

### Animals

*Cd157/Bst1* knockout (*Cd157*^−/−^) mice (C57BL/6J background) were previously described (Itoh et al., 1998) and maintained by crossbreeding homozygous mutant mice (Lopatina et al., 2014). Wild-type (*Cd157*^+/+^) and *Cd157*^−/−^ mice were kept in the animal center under standard conditions (24°C; 12-h light/dark cycle, lights on at 8:45 a.m.) in mouse cages (300 × 160 × 110 mm) with sawdust as bedding, and they received food and water *ad libitum*. Breeding pairs were separately maintained (1 pair per cage). At 21 days of age, offspring were removed, and housed in same-sex sibling pairs. Pups from postnatal days (PNDs) 3, 7, and 10 were used in this study.

Heterozygote *CD157*^+/−^ mouse pups were maintained by backcrossing *Cd157*^−/−^ with C57BL/6 mice. Offspring were genotyped as previously described (Itoh et al., 1998).

All animal experiments were conducted in accordance with the Fundamental Guidelines for the Proper Conduct of Animal Experiment and Related Activities in Academic Research Institutions under the jurisdiction of the Ministry of Education, Culture, Sports, Science, and Technology of Japan, and they were approved by the Committee on Animal Experimentation of Kanazawa University.

### Behavioral tests

The experimental animals were subjected to a series of behavioral tests performed 4 h before the dark phase. The mice were habituated to the room for 60 min before testing. The procedure for each behavioral test is described below. Dimensions of experimental chambers are represented as the (width × length × height). After each trial, the test chambers were cleaned with 80% alcohol and damp towels.

#### Ultrasonic vocalization recording

##### Isolation-induced USV recording in a mouse pup

Male C57BL/6, *Cd157*^+/−^, and *Cd157*^−/−^ pups (PND 3–10) from breeding pairs were tested as previously described (Liu et al., 2008, 2013a). Before testing, the cage with pups and their parents were transferred from the animal room to the test room for approximately 60 min for adaptaion. For pup isolation USVs, call recordings were performed in an anechoic box (700 × 600 × 600 mm) using the method described by Shu et al. (2005). Each pup was placed in a 500-ml glass beaker in the anechoic box. The microphone was located 5 cm above the pup for the 2-min recording period. Naïve mouse pups (*n* = 10–15) were used at every step of experiments to exclude pup handling and tiredness of infant mice.

##### Context-specific (courtship) USV recoding in adult mice

Male C57BL/6 and *Cd157*^−/−^ mice (10 weeks old, *n* = 13) were individually habituated to the testing environment for 30 min with a subsequent 5-min session pairing with an individual female. Vocalizations were recorded over a 5-min period.

##### Analysis of USVs

Recordings were performed blind to mouse genotype. Number of calls, and peak frequency (maximum syllable frequency point) and duration (difference between syllable start and end points) of emitted calls were measured using a USV monitor (SpectraLAB; Sound Technology Inc., Stage College, PA, USA; Liu et al., 2013a). Each syllable was classified as one of the following seven waveform categories: upward (up), downward (down), chevron, complex, harmonic, plate, or “V”-call. Classification was determined by internal pitch change, length, and shape, and was based on and adapted from previously described methods (Scattoni et al., 2008; Grimsley et al., 2011; Liu et al., 2013a; Zampieri et al., 2014). All syllable types exhibited typical structure from USV recorded spectrogram. Statistical analysis of peak frequency demonstrated a normal distribution pattern across data with relevant SD (data not shown). Thus, peak frequency and call duration were averaged across different syllable types. Click-like sounds of ≤40 ms in duration were filtered out of pup vocalizations (Liu et al., 2013a).

### Blood and tissue collection

All blood and tissue collection was performed independently of behavioral tests. Mice at different ages were anesthetized by intraperitoneal injection of pentobarbital (20 mg/kg body weight). Blood samples of 0.1–0.2 ml were collected by cardiac puncture and centrifuged at 1,600 × g for 15 min at 4°C. Plasma samples (~50–100 μl/mouse) were collected and stored at −80°C until use.

The whole hypothalamus was removed according to the stereotaxic coordinates (Franklin and Paxinos, 2008), and it was then homogenized in 10 mM Tris-base (pH 7.4) using a 1-ml Teflon/glass homogenizer. The fresh homogenates were used to determine ADP-ribosyl cyclase activity as previously described (Graeff et al., 1994; Liu et al., 2008; Lopatina et al., 2014). Protein content was determined using a Bio-Rad protein assay kit with bovine serum albumin as a standard (Bio-Rad, Hercules, CA, USA).

### Enzyme immunoassay for oxytocin

To determine the concentrations of OXT in the plasma, an OXT immunoassay kit was used according to the manufacturer's protocol (Assay Designs, Ann Arbor, MI) and as previously described (Jin et al., 2007; Zhong et al., 2016).

### ADP-ribosyl cyclase activity

Assessment of the ADP-ribosyl cyclase activity in the murine hypothalamus was performed in whole homogenates using the nicotinamide guanine dinucleotide technique as previously described (Graeff et al., 1994). Briefly, 2 ml of reaction mixtures containing 60 μM NGD^+^, 50 mM Tris-HCl (pH 6.6), 100 mM KCl, and 10 μM CaCl_2_ were maintained at 37°C with constant stirring. The samples were then excited at 300 nm, and fluorescence emission was continuously monitored at 410 nm with a Shimadzu RF-5300 PC spectrofluorometer (Kyoto, Japan). Activity was calculated from the linear portion of the 10-min time course by fitting a linear function to the data points recorded every 15 s. The specific ADP-ribosyl cyclase activity was calculated using cyclic GDP-ribose (cGDPR) standard, and the results are presented as nanomoles cGDPR per min per mg of protein (Jin et al., 2007; Lopatina et al., 2010).

### OXT treatment

OXT was injected (i.p.) in male pups (PNDs 7–10). Individual and group locomotor activities as well as isolation-induced USVs were recorded after 30, 60, 120, min of OXT administration, which was followed by blood plasma sampling.

### Statistical analysis

Data are expressed as mean ± SEM. The Kolmogorov-Smirnov test was used to examine cumulative sample distribution. Comparisons were made between two groups (*Cd157*^+/+^ and *Cd157*^−/−^) using two-tailed Student's *t*-test (for normal distributions) or Mann–Whitney *U*-test (absence of normal distribution). Two-way analysis of variance (ANOVA) was used to determine *Genotype* × *Age* or *Treatment* × *Call type* interactions. Subsequently, *post-hoc* Turkey's or Sidak's multiple comparison tests were used for group comparisons. Categorical variables were compared by χ^2^ test. In all analyses, *P* < 0.05 indicated statistical significance.

## Results

**Figure 1:**
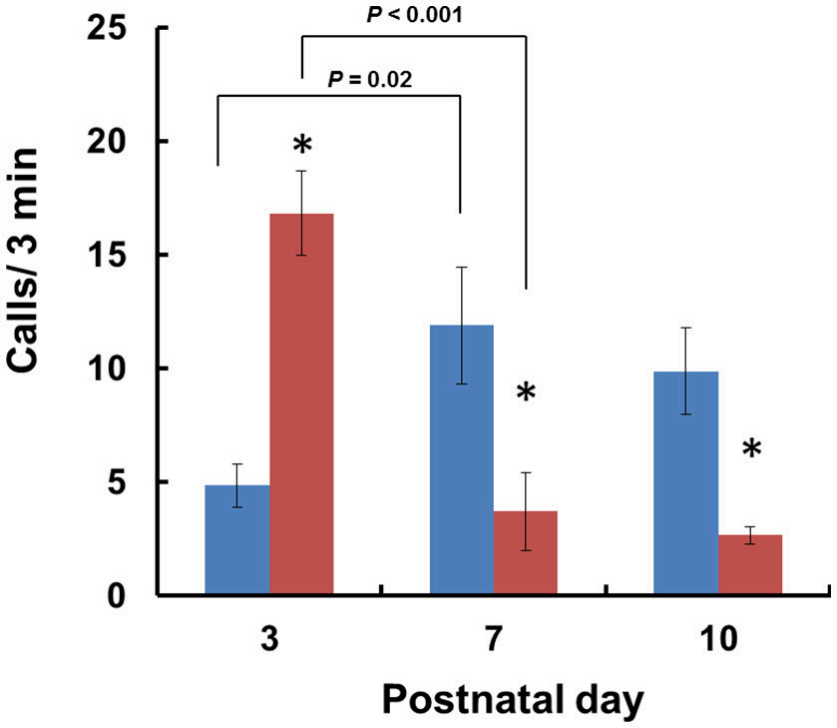
**Development of isolation-induced USV production in ***Cd157***^**+/+**^ and ***Cd157***^**−/−**^ mouse pups**. The number (*n* = 10–15) of ultrasonic calls were measured in wild-type (*C57BL/6*: blue) and *Cd157*^−/−^ (red) pups at PNDs 3–10. Data are given as the means ± S.E.M. Two-way ANOVA with the *post-hoc* Tukey's multiple comparison test was evaluated. ^*^P < 0.05 from *Cd157*^+/+^ pups.

When isolated from their mothers, mouse pups produce USVs until PND 12 (Scattoni et al., 2009). Consequently, we examined isolation-induced USVs in *Cd157*^+/+^ and *Cd157*^−/−^ pups at PNDs 3, 7, and 10. Two-way ANOVA demonstrated a significant *Genotype* × *Age* interaction [*P* < 0.0001, *F*_(2, 14)_ = 17.54, *n* = 10–15]. Analysis of USVs by *post-hoc* Turkey's multiple comparison test showed a significantly higher call number at PND 3 in *Cd157*^−/−^ mice compared with *Cd157*^+/+^ controls (17 ± 2 calls/min vs. 5 ± 1 calls/min per 3-min session, respectively; *P* < 0.001, *n* = 10–15; Figure 1). During the first postnatal week, the number of USVs in *Cd157*^−/−^ mice dramatically decreased to 4 ± 2 calls/min at PND 10 (*P* = 0.02, χ^2^ = 0.974, *n* = 10–15). By contrast, the number of USVs in *Cd157*^+/+^ mice increased at PNDs 7 and 10: 12 ± 3 and 10 ± 2 calls per 3-min session, respectively, from 5 ± 1 calls at PND 3 (Figure 1). The number of USVs in *Cd157*^−/−^ mice at PNDs 7 and 10 was higher than in *Cd157*^−/−^ mice (*P* < 0.0001, *n* = 10–15). Supplementary Table 1 summarizes call type and number produced by *Cd157*^+/+^ and *Cd157*^−/−^ mice at PNDs 3, 7, and 10.

Frequency of USVs decreased equally in both genotypes during development from PNDs 3–10, with no significant *Genotype* × *Age* interaction [two-way ANOVA: *P* < 0.1893, *F*_(2, 74)_ = 1.681, *n* = 10–1]. Indeed, there were no differences between *Cd157*^+/+^ and *Cd157*^−/−^ mice, except for lower USV frequency at PND 7 in *Cd157*^+/+^ mice (61.1 ± 1.1 kHz) compared with *Cd157*^−/−^ mice (65.0 ± 1.2 kHz; *post-hoc* Turkey's multiple comparison test: *P* = 0.02, χ^2^ = 0.7778; Supplementary Figure 1A). Duration of USVs was essentially unchanged in *Cd157*^+/+^ mice during development (Supplementary Figure 1B), although two-way ANOVA showed a significant *Genotype* × *Age* interaction [*P* < 0.0001, *F*_(2, 84)_ = 45.10, *n* = 10–15; *Genotype, P* < 0.0001, *F*_(1, 84)_ = 25.4; *Age, P* < 0.0001, *F*_(2, 84)_ = 34.76]. At PND 3, duration of USVs was extremely high in *Cd157*^−/−^ mice (86.2 ± 4.3 ms) compared with *Cd157*^+/+^ mice (52.8 ± 1.8 ms) (*post-hoc* Turkey's multiple comparison test: *P* < 0.0001). During the first postnatal week, USV duration in *Cd157*^−/−^ mice reduced to 49.8 ± 1.5 ms (PND 7). Consistent with a previous report (Scattoni et al., 2009), neither genotype emitted isolation-induced USVs after PND 10.

**Figure 2:**
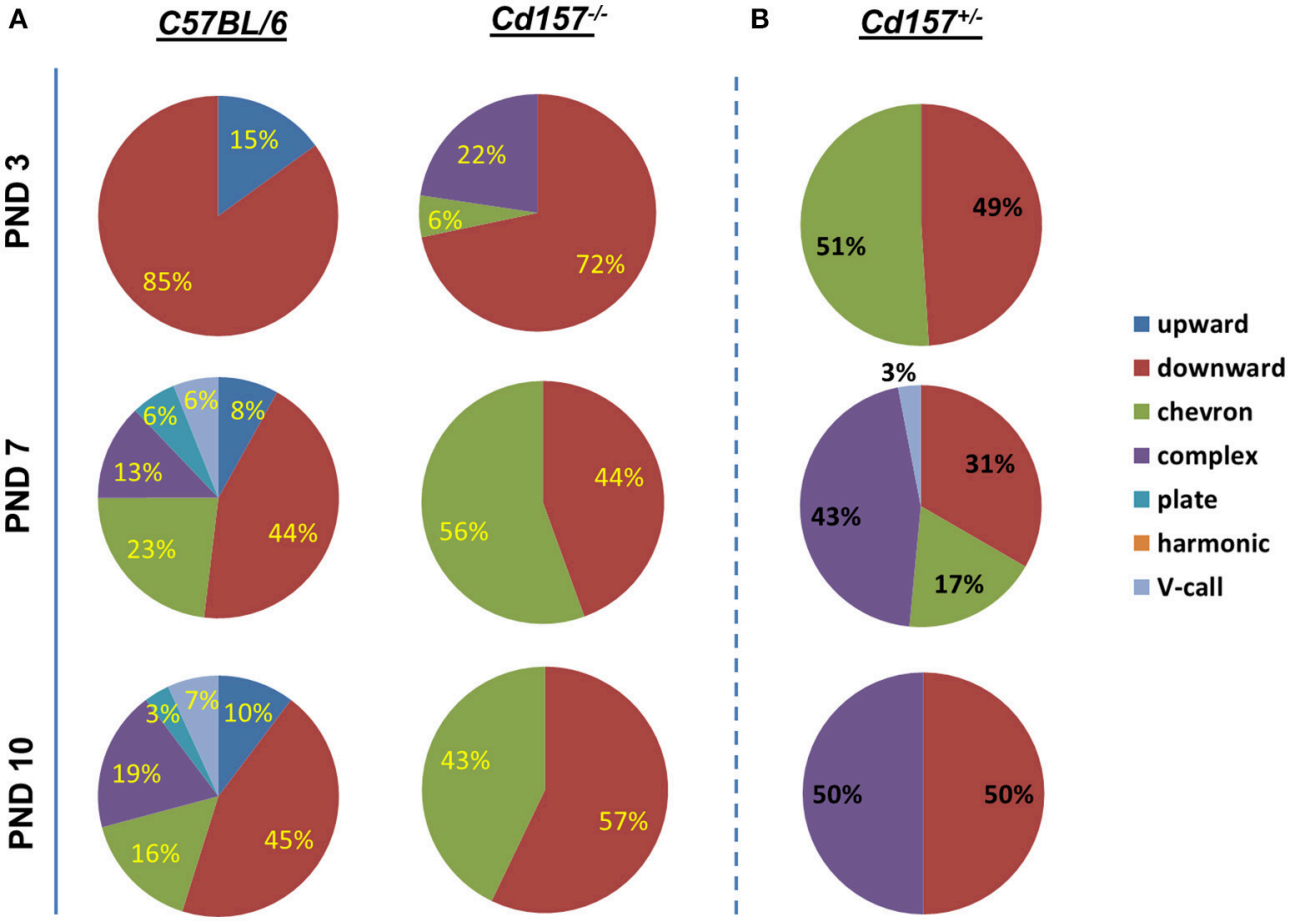
**Call category variations in USVs emitted pups with Cd157 gene deletion at PNDs 3, 7, and 10**. Percentages of call syllable were calculated in *Cd157*^+/+^ **(A)**, *Cd157*^−/−^ **(A)**, and *Cd157*^+/−^ **(B)** mouse groups as the number of calls in each category for each subject/total number of calls analyzed in each subject. (Number of pups used = 15–20, number of calls analyzed = 100–120). The calculated data of calls are presented in the Supplementary Table 1.

Voice patterns of USVs from *Cd157*^+/+^ and *Cd157*^−/−^ mice in the first two postnatal weeks are shown in Figure 2A. Three-day-old *Cd157*^−/−^ pups emitted USV calls consisting of three categories (72% downwards, 22% upwards, and 6% chevron calls), whereas 3-day-old *Cd157*^+/+^ pups demonstrated similar waveform patterns but consisting of only two call types (15% upward and 85% downward). Two-way ANOVA demonstrated a significant *Genotype* × *Call type* interaction at PND 7 [*P* < 0.0001, *F*_(2, 114)_ = 22.39, *n* = 10–15]. However, interestingly, at PND 7, *Cd157*^+/+^ mice emitted a broader, multi-faceted repertoire of USVs, which already contained six call types (44% downward, 23% chevron, 13% complex, 8% upward, 6% plate, and 6% “V” calls). In contrast, *Cd157*^−/−^ pups displayed no complexity in USV categories, and only emitted two USV call types (44% downward and 58% chevron). Two-way ANOVA detected a significant *Genotype* × *Call type* interaction at PND 7 [*P* < 0.0001, *F*_(6, 266)_ = 45.18, *n* = 10–15]. This USV pattern of a rich repertoire in *Cd157*^+/+^ mice and poor repertoire in *Cd157*^−/−^ pups continued from PND 7 until 10, with two-way ANOVA confirming a significant *Genotype* × *Call type* interaction at PND 10 [*P* < 0.0001, *F*_(6, 266)_ = 44.69, *n* = 10–15]. Detailed inspection found that 7-day-old *Cd157*^−/−^ pups emitted a higher percentage of chevron calls [*Cd157*^+/+^ (23%) vs. *Cd157*^−/−^ (56%), *P* < 0.001, *n* = 10–15], but the same percentage of downward voice events (44 and 43% for both genotypes, respectively). Thus, while USV repertoire developed in wild-type pups, there was no developmental progress in knockout pups, and instead “retardation” was apparent. To investigate gene-dosage effects on USV repertoire, we examined isolation-induced USVs in heterozygote (*Cd157*^+/−^) infant males at PNDs 3, 7, and 10 (Supplementary Table 1). *Cd157*^+/−^ pups displayed intermediate patterns of USVs, in which no clear development of complex patterns with varied voice types was observed, although 3–4 voice types were detected (Figure 2B).

**Figure 3:**
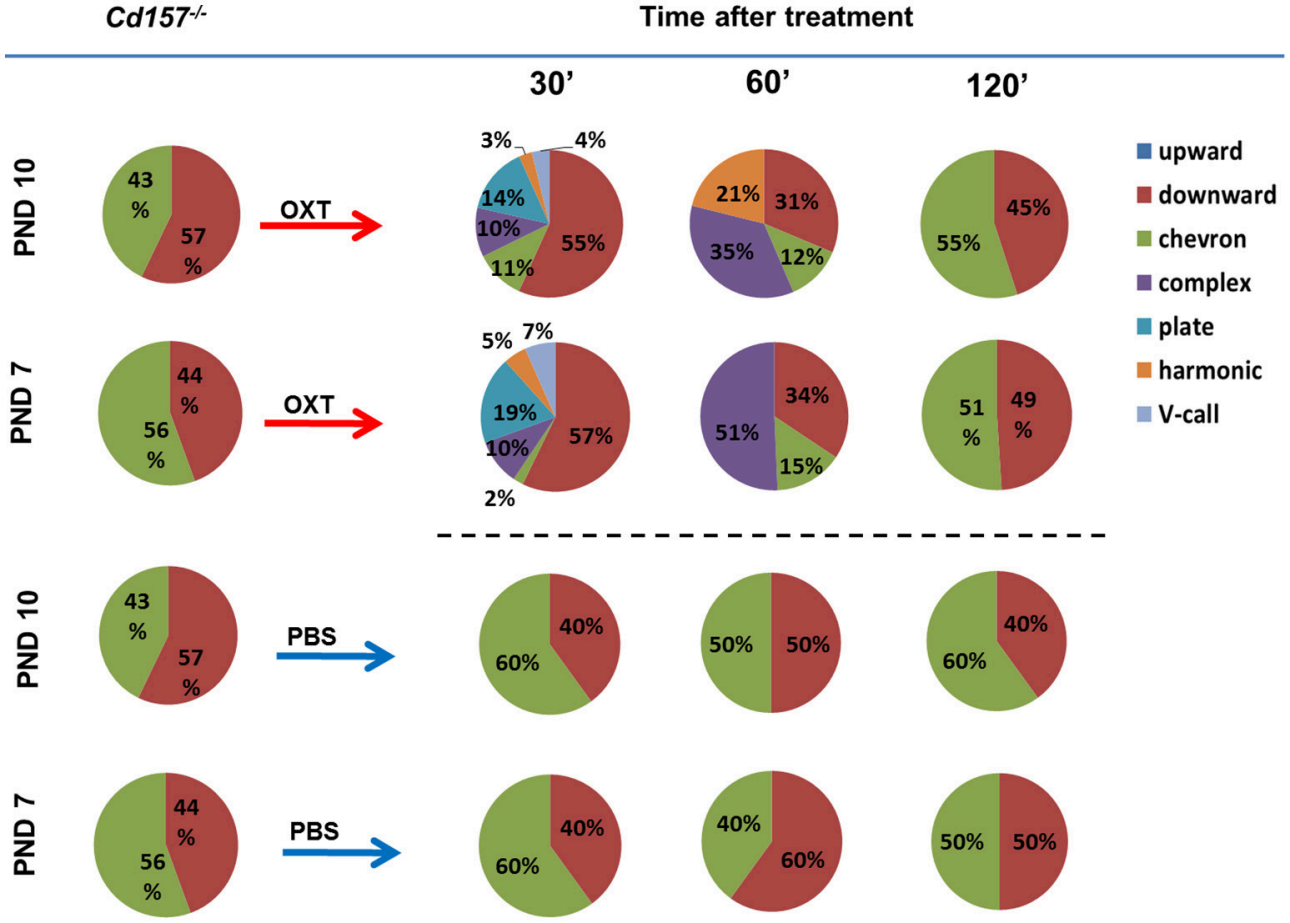
**Oxytocin treatment affects the USV pattern in ***Cd157***^**−/−**^ pups at PNDs 7 and 10**. Call category variations of USVs emitted by mouse pups at PNDs 7 and 10 were classified after 30, 60 and 120 min of intraperitoneal injection of OXT (10 ng/mouse) or PBS. Percentages were calculated as the number of calls in each category for each subject/total number of calls analyzed in each subject (Number of pups tested = 10–15; number of calls analyzed = 80–100). The calculated data of calls are presented in the Supplementary Tables 2, 3.

Next, we sought to determine whether OXT facilitates USV development and can influence the observed retardation at PNDs 7 and 10. Within 30 min, intraperitoneal OXT injection (10 ng/mouse) triggered a dramatically richer USV repertoire, with two-way ANOVA confirming a significant *Treatment* × *Call type* interaction [*P* < 0.001, *F*_(2, 266)_ = 73.53, *n* = 10–15] (Figure 3, Supplementary Tables 2, 3). At 60 min after OXT injection, USV pattern reduced to 3 or 4 voice types (from six types at 30 min). At 120 min, the repertoire returned to the initial pattern of two voice types. Thus, OXT clearly influences the normal wild-type pattern, but the effect is transient, and OXT cannot facilitate development nor fundamentally alter voice repertoire.

PBS injection had no effect on USV repertoire in *Cd157*^−/−^ pups at PNDs 7 or 10 (Figure 3, Supplementary Tables 2, 3). Intraperitoneal OXT injection (10 ng/mouse) in *Cd157*^+/−^ at PNDs 7 and 10 strikingly altered the rich USV repertoire within 30 min, which returned to initial levels (two voice types in 120 min), similar to *Cd157*^−/−^ mice (data not shown).

**Figure 4:**
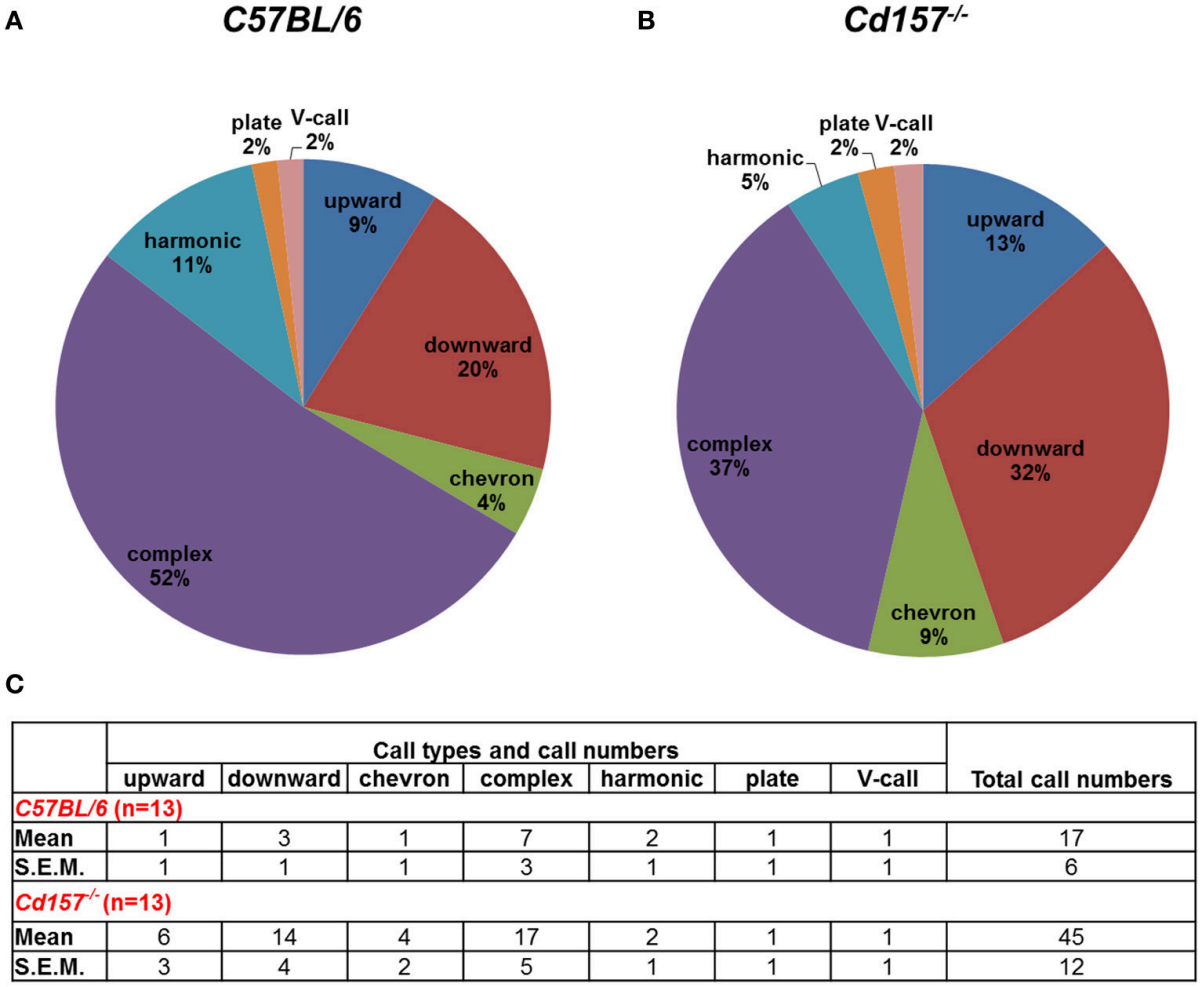
**Call category outline of USVs in adult ***Cd157***^**+/+**^ and ***Cd157***^**−/−**^ male mice**. A wild-type (*C57BL/6*, **A**) or *Cd157*^−/−^ **(B)** mouse was placed with a virgin female stranger mouse. USVs recorded during courtship were calculated as the number of calls in each category for each subject/total number of calls analyzed in each subject as a percentage. The recorded data of calls are presented in the **(C)**. The number of pairs tested = 8–10 and number of calls analyzed = 100–120.

Adult male mice do not produce USVs under social isolation. Consequently, to assess vocalization ability in adult mice, we measured USV parameters during courtship, because many USVs are emitted in the context of sexual behavior from male mice to females. With these USV recordings, *Cd157*^−/−^ mice produced more calls in comparison to *Cd157*^+/+^ mice (45 ± 12 vs. 17 ± 6 respectively, *P* < 0.05, *n* = 10–15; Figure 4). However, *Cd157*^−/−^ mice emitted USVs with a rich repertoire (seven different voice types), similar to *Cd157*^+/+^ mice (also seven types). We did not detect any difference in peak frequency or call duration between genotypes (data not shown). These results show that *Cd157*^−/−^ mice can emit “normal” USVs, and therefore do not possess a genetic defect in voice emissions. Our results also show that *Cd157*^−/−^ pups with restricted USV patterns show “a” developmental delay that is “corrected” in adult ages when seeking a partner.

**Figure 5:**
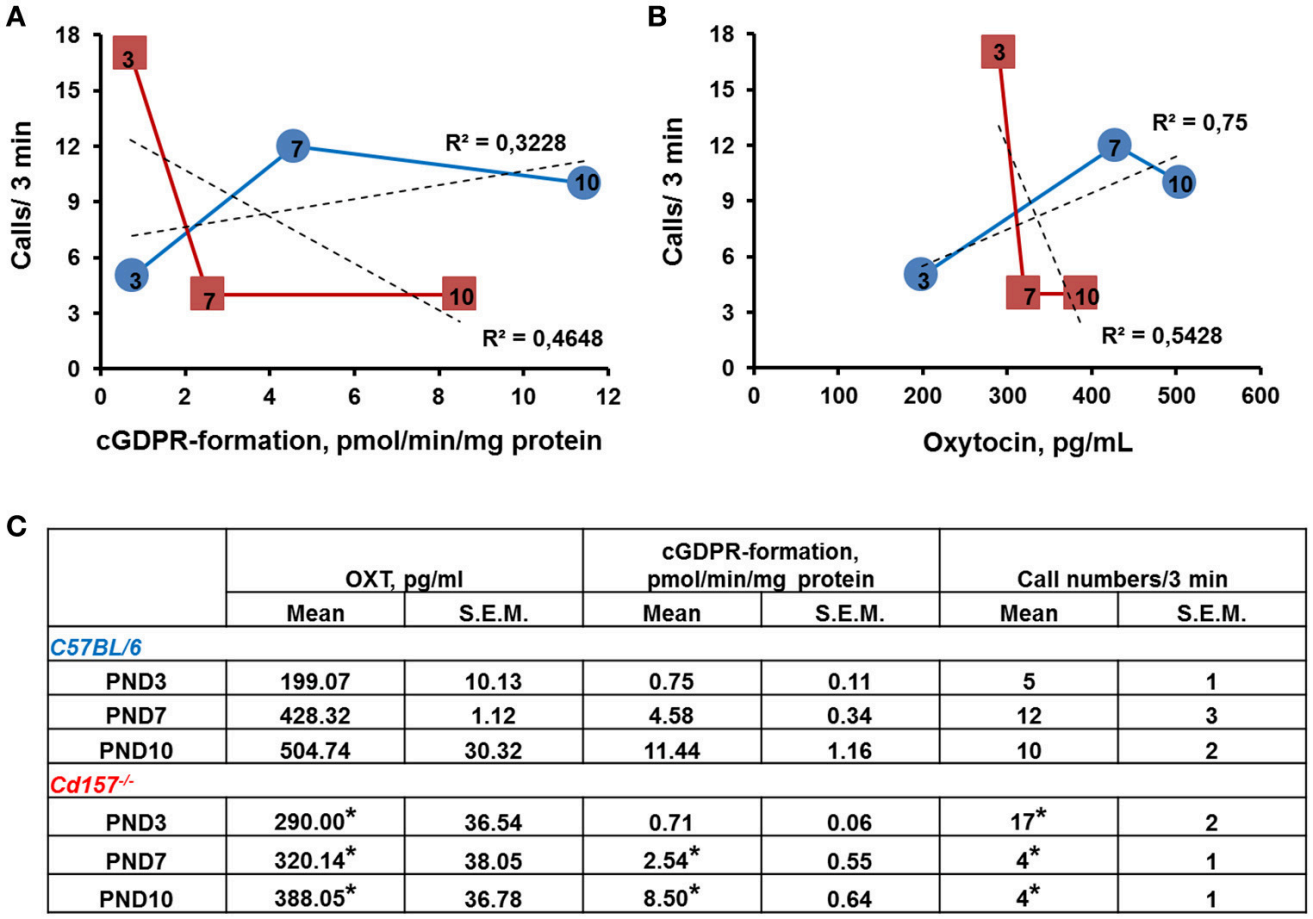
**ADP-ribosyl cyclase activity and plasma concentration of oxytocin in correlation with call number in ***Cd157***^**+/+**^ and ***Cd157***^**−/−**^ mice during 10 days of life. (A)** ADP-ribosyl cyclase activity in relation to call number in *Cd157*^+/+^ (blue round) and *Cd157*^−/−^ (red squares) mice at post-natal days (PNDs) 3, 7, and 10. ADP-ribosyl cyclase activity was measured as rate of cyclic GDP-ribose formation in whole-cell homogenates isolated from the hypothalamus. **(B)** Plasma oxytocin (OXT) concentration correlates with call number in *Cd157*^+/+^ (blue round) and *Cd157*^−/−^ (red squares) mice at PNDs 3, 7, and 10. Data were obtained from pups at the indicated ages (arabic numbers). **(C)** Recorded ADP-ribosyl cyclase activity, plasma concentration of OXT, and call number in *Cd157*^+/+^ and *Cd157*^−/−^ mice at PNDs 3, 7, and 10. ^*^P < 0.05 from *Cd157*^+/+^ pups at the corresponding measuring day.

To determine whether the observed alterations in USVs might be linked to impaired OXT secretion (because of *Cd157* deletion), we examined ADP-ribosyl cyclase activity, which is required for central OXT secretion (Jin et al., 2007) in the hypothalamus, as well as OXT plasma levels in *Cd157* wild-type and knockout mice during the first 10 days of life (Figure 5). Two-way ANOVA detected a non-significant *Genotype* × *Age* interaction in ADP-ribosyl cyclase activity [*P* = 0.0600, *F*_(2, 24)_ = 3.17, *n* = 10–15]. However, significant differences separately influenced by *Genotype* [*P* = 0.0025, *F*_(1, 24)_ = 11.37] and *Age* [*P* = 0.0001, *F*_(2, 24)_ = 123.4] were detected. *Post-hoc* Sidak's multiple comparison test confirmed significantly lower hypothalamic ADP-ribosyl cyclase activity at PND 7 (*P* = 0.018, *n* = 10–15) and PND 10 (*P* = 0.003, *n* = 10–15) in *Cd157*^−/−^ mice compared with *Cd157*^+/+^ mice. Two-way ANOVA also showed a significant *Genotype* × *Age* interaction in plasma OXT levels [*P* = 0.045, *F*_(2, 35)_ = 3.386, *n* = 10–15], with lower plasma OXT levels in knockout mice at PND 7 [*P* = 0.033, *n* = 10–15] and PND 10 (*P* = 0.04, *n* = 10–15] (*post-hoc* Sidak's multiple comparison test). ADP-ribosyl cyclase activity and plasma OXT levels reversed, and starting from PND 7, we observed lower ADP-ribosyl cyclase activity and plasma OXT levels in *Cd157*^−/−^ mice compared with wild-type mice (*P* < 0.05). In *Cd157*^+/+^ mice, call number was positively correlated with higher ADP-ribosyl cyclase activity (*R*^2^ = 0.3228; Figure 5A) and plasma OXT levels (*R*^2^ = 0.75; Figure 5B), according to age. In contrast, *Cd157*^−/−^ mice demonstrated negative correlation between emitted call number and ADP-ribosyl cyclase activity (*R*^2^ = 0.4648; Figure 5A) and plasma OXT (*R*^2^ = 0.5428; Figure 5B) over PNDs 3–10.

USV characterization and plasma OXT levels after OXT administration in *Cd157*^−/−^ mice at PND 7 are listed in Table 1. At 30 and 60 min after OXT application, USV features were altered and showed a significant difference compared with PBS application. Hence, it is likely that changes in USV reflect shifting plasma OXT levels.

**Table 1:** **USV characterization and plasma OXT level after OXT administration in ***Cd157***^**−/−**^ pups**.

***Cd157*^−/−^, PND7**	**Drug**	**Time after drug administration (i.p.), min**			
		**0′**	**30′**	**60′**	**120′**
Calls/3min (*n* = 8–14)	Control	3 ± 2			
	PBS		5 ± 2	4 ± 2^*^	4 ± 2
	OXT		4 ± 1	9 ± 1	5 ± 1
Frequency, kHz (*n* = 18)	Control	65.0 ± 1.3			
	PBS		$\begin{array}{cc} \begin{array}{l} 67.1\pm 1.5\\ 56.4\pm 2.7 \end{array} & ] \end{array}$^*^	$\begin{array}{cc} \begin{array}{l} 66.4\pm 2.3\\ 54.7\pm 2.3 \end{array} & ] \end{array}$^*^	64.3 ± 3.1
	OXT				63.3 ± 2.2
Duration, ms (*n* = 18)	Control	49.7 ± 2.1			
	PBS		$\begin{array}{cc} \begin{array}{l} 51.3\pm 2.1\\ 57.1\pm 0.9 \end{array} & ] \end{array}$^*^	$\begin{array}{cc} \begin{array}{l} 50.9\pm 2.5\\ 59.5\pm 0.8 \end{array} & ] \end{array}$^*^	50.3 ± 1.8
	OXT				53.3 ± 3.2
Plasma OXT level, pg/ml (*n* = 8–14)	Control	318.4 ± 37.9			
	PBS		$\begin{array}{cc} \begin{array}{l} 312.5\pm 33.3\\ 646.9\pm 84.6 \end{array} & ] \end{array}$^*^	$\begin{array}{cc} \begin{array}{l} 320.7\pm 27.8\\ 549.4\pm 24.9 \end{array} & ] \end{array}$^*^	323.7 ± 19.5
	OXT				346 ± 32.8

* *p < 0.001*.

## Discussion

Using a mouse model of neurodevelopmental disease to study social communication and interaction is an important strategy for shedding light on the black box of molecular processes that underlie formation and implementation of social behavior, as well as aiding identification of new molecular targets for pharmacological treatment of communication impairments. Here, we demonstrated that *Cd157* knockout mice have developmental abnormalities, and do not exhibit the normal, full range of USVs during the lactating period.

Mice emit USVs under different social conditions throughout their lifespan (Scattoni et al., 2009). Pups separated from the nest and their mother emit vocalization signals with clear communicative value, which correlate with social approach or exploratory behavior (D'amato, 1991; Scattoni et al., 2008). Since the first description by Zippelius and Schleidt (1956), neonatal USVs have been interpreted as a communicative behavior (Wöhr and Schwarting, 2007; Hammerschmidt et al., 2009; Scattoni et al., 2009; Shepard and Liu, 2011). Neonatal USVs also map onto later development of adult anxiety profiles (Dichter et al., 1996; Scattoni et al., 2009). Consequently, loss of USV complexity after PND 3, as observed in *Cd157*^−/−^ mice, may reflect delayed development of communication skills. Moreover, it may be partially associated with the pre-existing autistic- (anxiety- and avoidance-like) behavior that has already been reported in *Cd157*^−/−^ mice (Lopatina et al., 2014).

It is particularly intriguing that adult knockout mice show a full, complex syllable repertoire, whereas juveniles do not, unless under exogenous OXT administration. This begs the question of when exactly juveniles develop the ability to produce these other vocalizations in a more natural context. As the mother-pup social interaction is most important in mice, isolation from dams initiates USV emission (Scattoni et al., 2009). Nonetheless, it is also well-known that isolation from dams to a cold location causes pups to intensively emit very distinct USVs of the complex type (Branchi et al., 2001). This type of alarm call was indistinguishable between pups of the different genotypes (data not shown). Therefore, a delay in variety of syllable patterns relates more to social interaction-based maturation, because life-threatening-based USVs are the same.

Vocal delay was restored at the adult age when seeking a partner, suggesting that any delay is likely corrected in adults. Estimation that this correction is undertaken during the juvenile period later than PND 10 is possible, but was not tested in the current experiments. This question “cannot be tested” because it is known that isolation-induced USVs sharply decrease after PND 10, during the late suckling period (Branchi et al., 2001). At best, we can state that this delay is not apparent in adults in the context of partner seeking.

The OXT neurotransmitter system regulates USV signaling in rodents, and has been previously reviewed (Branchi et al., 2001). We found that communicative ability of wild-type pups corresponded to higher plasma OXT levels and elevated ADP-ribosyl cyclase activity in the hypothalamus, while preserved communicative ability of *Cd157*^−/−^ pups was not positively associated with either variable with age. In fact, both values were apparently strain-specific. At PND 7, OXT administration restored communication skills and plasma OXT levels in *CD157*^−/−^ mice, but had no effect in wild-type mice. Further, these strain differences in ADP-ribosyl cyclase activity and OXT levels are indirect, and only show a developmental profile of the two variables. Accordingly, our findings do not provide evidence that these two variables are related in a causative way, or to vocal behavior.

Exogenous OXT administration in isolated rat pups reduces the rate of USVs, although OXT antagonist treatment does not change USVs (Insel and Winslow, 1991), and OXT null mutant mouse pups display fewer USVs than wild-type controls (Winslow et al., 2000). This counterintuitive finding (given that exogenous OXT also decreases calling) has been interpreted as evidence that social separation is not perceived as distress, and does not induce USVs in the absence of OXT (Winslow et al., 2000; Winslow and Insel, 2002; Scattoni et al., 2009). Takayanagi et al. (2005) reported that OXT receptor knockout pups exhibited fewer USVs at 7-days-old, which is consistent with the hypothesis that OXT neurotransmission is necessary for perception of social separation and subsequent USV response. In our current experiments, ADP-ribosyl cyclase-mediated OXT release was altered by PND 7 in *Cd157*^−/−^ mice, resulting in aberrant USVs. Interestingly, this effect was prevented by exogenous OXT administration. Notably, our previous observations (Liu et al., 2008) demonstrated that *Cd38* knockout mice had abnormal vocalizations during the neonatal period, while 7-day-old *Cd38* knockout pups (compared with *Cd38* wild-type mice) expressed significantly lower levels of hypothalamic and neurohypophysial ADP-ribosyl cyclase activity.

CD157 and CD38 have similar amino acid sequences and ADP-ribosyl cyclase activity (catalyzing cyclic ADP ribose), but distinct NAD base exchange activity (producing nicotinic acid adenine dinucleotide phosphate; Higashida et al., 2017). Further, both are involved in NAD^+^ metabolism (Lee, 2012; Higashida et al., 2017). Additionally, the human *CD157* gene shares unique genomic organization with the human *CD38* gene (Ortolan et al., 2002). Taking into consideration that CD38 and CD157 belong to the same family of NAD^+^-glycohydrolases (Lee, 2001; Guse, 2005), it is tempting to speculate that CD157 might be involved in the mechanism of OXT release in a similar manner as CD38. However, this needs to be clarified in relation to developmental delay in future experiments.

Previously, we found that CD157 and CD38 and their signaling pathways are shared in anxiety, autism, early atypical motor function, speech and language disorders, and social avoidance (Lopatina et al., 2014). Although we demonstrated differences between *Cd38* and *Cd157* knockout mouse behavior (even on different mouse backgrounds; Jin et al., 2007; Lopatina et al., 2014; Higashida et al., 2017), the exact role of CD157 in OXT secretion and regulation of social behavior requires further investigation. One suggested approach would be to generate *Cd38/Cd157* double knockout mice and confirm the compensatory role of *Cd157* in *Cd38* knockouts.

In summary, this is the first study to demonstrate association of CD157 with neonatal developmental delay in communicative ability, which could be rescued by exogenous OXT administration. Additionally, the *CD157* gene may be a valuable candidate for involvement in increased risk for anxiety or social avoidance. Our data may have important implications for future studies of anxiety disorders, social avoidance, and communication delay in children with ASD, which can slowly develop with age.

## Author contributions

OL and HH conceived and designed the research. All performed experiments on mice. OL and KF analyzed data and prepared figures. OL and AS prepared the initial draft; OL, AS, and HH revised the manuscript. All authors reviewed the final manuscript and approved its publication.

### Conflict of interest statement

The authors declare that the research was conducted in the absence of any commercial or financial relationships that could be construed as a potential conflict of interest. The reviewer DT and handling Editor declared their shared affiliation, and the handling Editor states that the process nevertheless met the standards of a fair and objective review.

## Supplementary material

Supplementary Figure 1 **Development of isolation-induced USV production in *Cd157*^+/+^ and *Cd157*^−/−^ mouse pups**. Frequency **(A)** (*n* = 18) and duration **(B)** (*n* = 18) of ultrasonic calls were measured in wild-type (C57BL/6) and *Cd157*^−/−^ pups at post-natal days (PNDs) 3–10. Data are shown as mean ± SEM. Two-way ANOVA with *post-hoc* Tukey's multiple comparison test was performed. ^*^*P* < 0.05 from *Cd157*^+/+^ pups.

Supplementary Table 1 **Call types and call numbers C57BL/6, Cd157^−/−^, *Cd157*^+/−^ male mice at PND3,7,10**.

Supplementary Table 2 **Call types and call numbers after PBS or oxytocin (OXT) treatment in *Cd157*^−/−^ male mice at PND7**.

Supplementary Table 3 **Call types and call numbers after PBS or oxytocin (OXT) treatment in *Cd157*^−/−^ male mice at PND10**.
