# Supplementary figures and images for: Communication Impairment in Ultrasonic Vocal Repertoire during the Suckling Period of Cd157 Knockout Mice: Transient Improvement by Oxytocin
Source: Front Neurosci. 2017 May 17;11:266. doi: 10.3389/fnins.2017.00266 (PMC5434149; doi:10.3389/fnins.2017.00266)

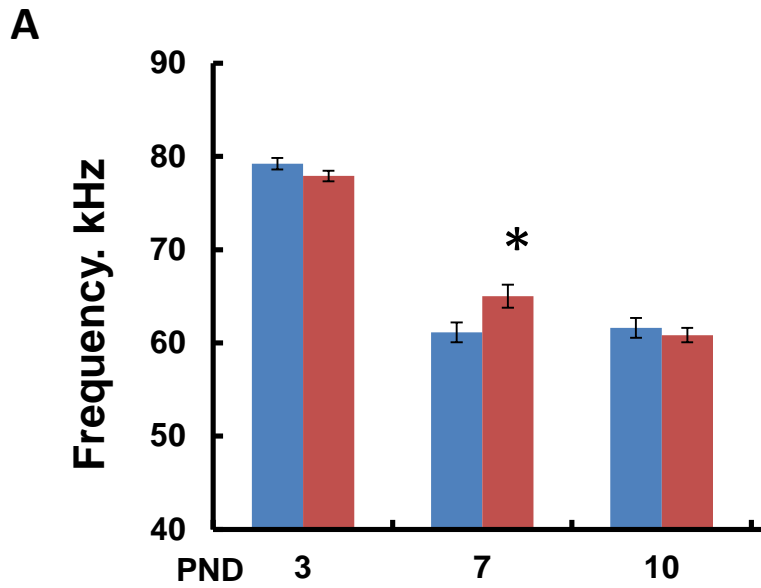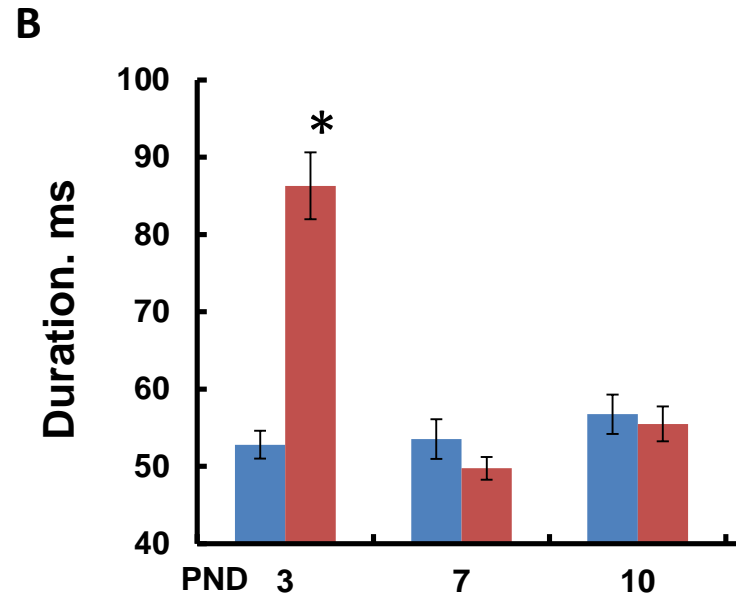

**Supplementary Figure 1**

Supplement: Supplementary Figure 1 — Development of isolation-induced USV production in Cd157+/+ and Cd157−/− mouse pups. Frequency (A) (n = 18) and duration (B) (n = 18) of ultrasonic calls were measured in wild-type (C57BL/6) and Cd157−/− pups at post-natal days (PNDs) 3–10. Data are shown as mean ± SEM. Two-way ANOVA with post-hoc Tukey's multiple comparison test was performed. *P < 0.05 from Cd157+/+ pups. [file Image1.PDF]

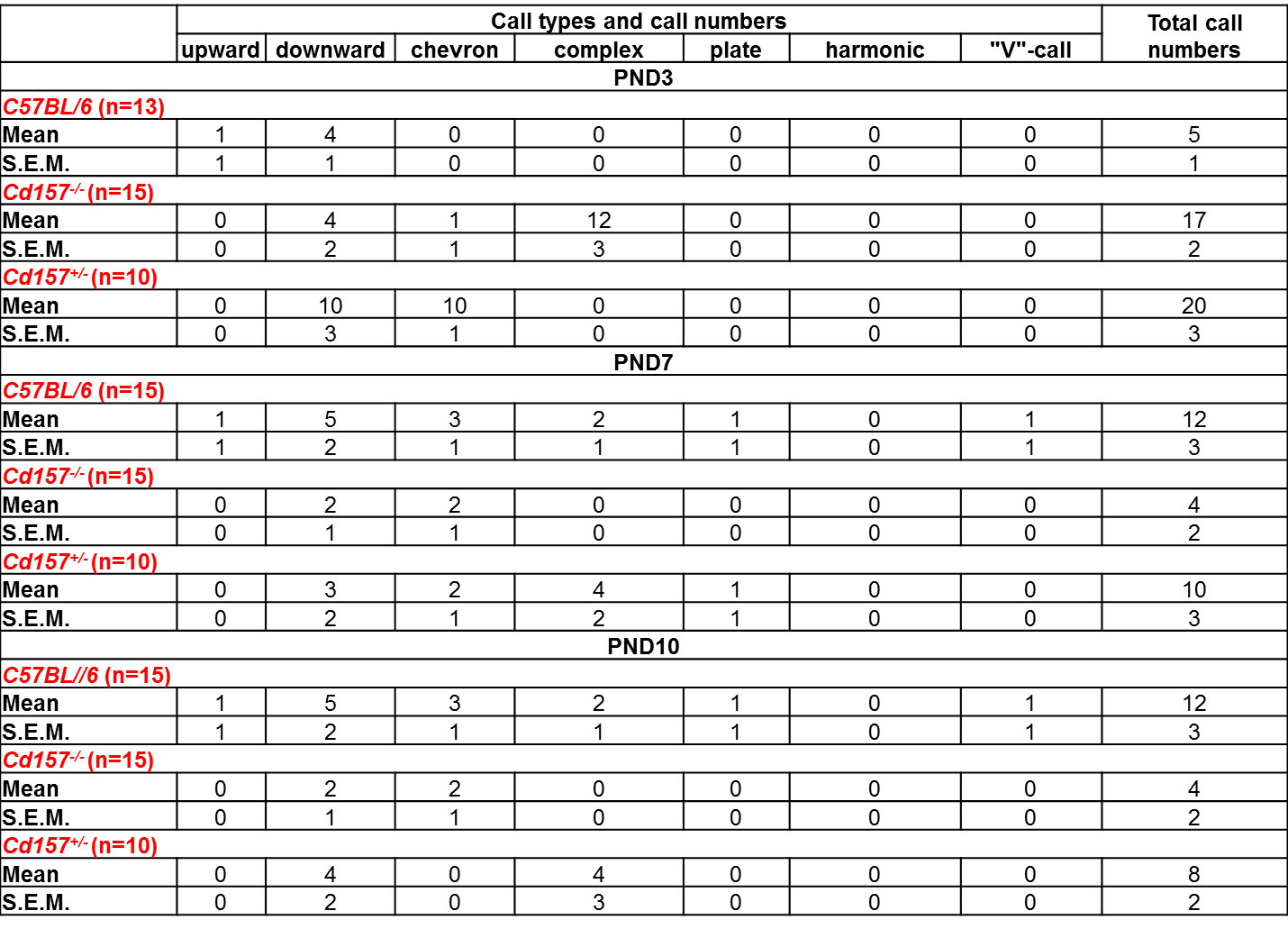


**Supplementary Table 1.**

**Call types and call numbers C57BL/6, *Cd157-/- , Cd157+/-* male mice at PND3,7,10.**

Supplement: Supplementary Table 1 — Call types and call numbers C57BL/6, Cd157−/−, Cd157+/− male mice at PND3,7,10. [file Table1.DOCX]
