# Supplementary material for: Communication Impairment in Ultrasonic Vocal Repertoire during the Suckling Period of Cd157 Knockout Mice: Transient Improvement by Oxytocin
Source: Front Neurosci. 2017 May 17;11:266. doi: 10.3389/fnins.2017.00266 (PMC5434149; doi:10.3389/fnins.2017.00266)
Supplement: Supplementary Table 3 — Call types and call numbers after PBS or oxytocin (OXT) treatment in Cd157−/− male mice at PND10. [file Table3.DOCX]

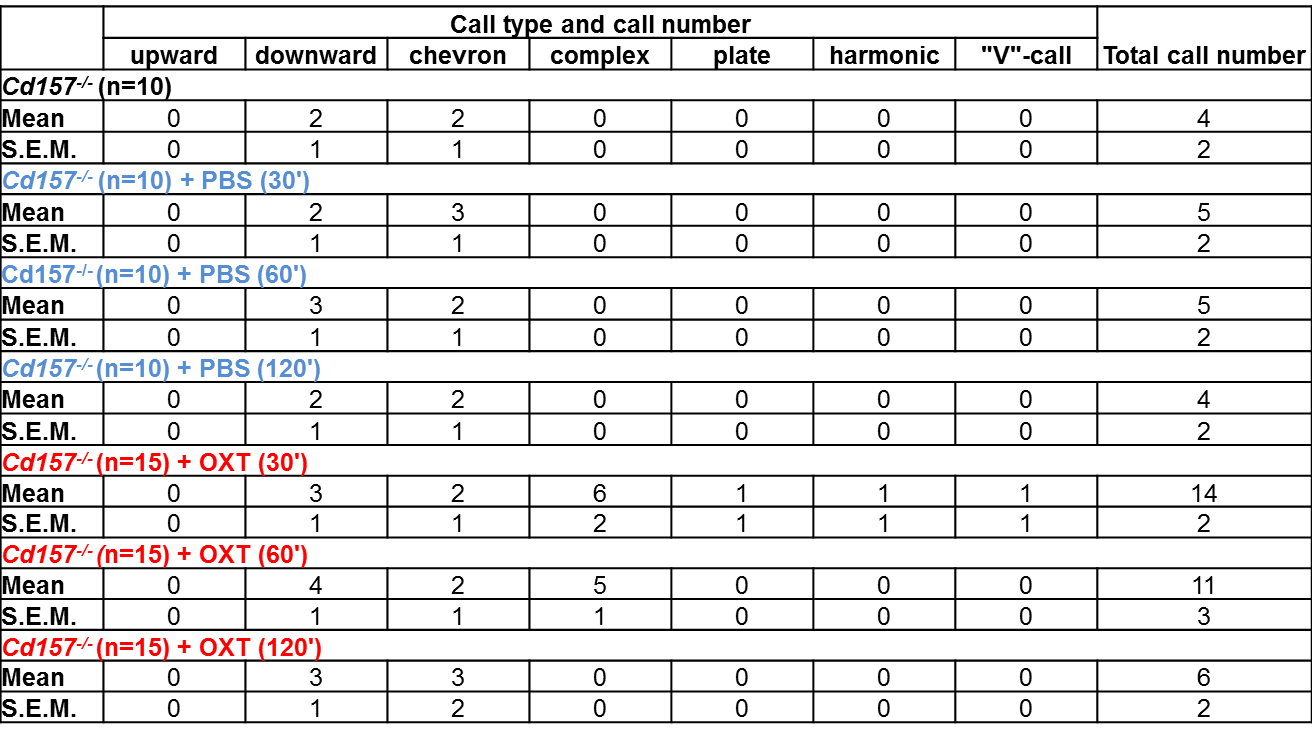


**Supplementary Table 3.**

**Call types and call numbers after PBS or oxytocin (OXT) treatment in *Cd157^-/-^* male mice at PND10.**
